# Polyurethane Foam and Algae-Based Activated Carbon Biocomposites for Oil Spill Remediation

**DOI:** 10.3390/ma17164137

**Published:** 2024-08-21

**Authors:** Lokmane Abdelkaddous Baidar, Malika Medjahdi, Badra Mahida, Belaid Mechab, Dominique Baillis

**Affiliations:** 1 GCE Laboratory, Sidi bel Abbes 22000, Algeria; lokmanbaidar17@gmail.com; 2 APELEC Laboratory, Sidi bel Abbes 22000, Algeria; 3 LaRTFM Laboratory, Oran 31000, Algeria; badra_mahida@yahoo.fr; 4 LMPM Laboratory, Sidi bel Abbes 22000, Algeria; bmechab@yahoo.fr; 5 LaMCoS, INSA-Lyon, CNRS UMR5259, 69621 Villeurbanne, France; dominique.baillis@insa-lyon.fr

**Keywords:** polyurethane foam, oil spill, biocomposite, cleanup

## Abstract

This study investigates the incorporation of algae-based activated carbon into polyurethane foam to improve a biocomposite for gasoil sorption. The biocomposites were thoroughly analyzed using various techniques to examine the properties of both the blank foam and the algae activated carbon foam with a carbon content of 4.41 mass% and particle diameter of 500 µm. These techniques included Scanning Electron Microscopy (SEM), thermogravimetric analysis (TGA), and density analysis. The TGA analysis revealed that the biocomposites had an impact on the onset temperature (T_onset_) of the foams. Higher concentrations of the biocomposites resulted in a decrease in T_onset_ from approximately 310 °C in the blank foam (PUF0) to 300 °C in the composite (PUF3B). The final residue percentage also decreased from around 20% in PUF0 to 10% in PUF3B. Density analysis showed that the apparent density of the foam increased from 0.016 g/cm^3^ in the blank foam to 0.020 g/cm^3^ in the biocomposite (PUF3B), while the real density slightly decreased from 0.092 g/cm^3^ to 0.076 g/cm^3^, indicating a reduction in overall porosity from 82.5% to 74.4%. All foams that were modified showed an increase in their ability to absorb gasoil in a PUF/gasoil/water system. The optimized biocomposite (PUF1B), with 1.14 mass% of 500 µm algae carbon, displayed the highest sorption capacity, starting at approximately 50 g/g at 1.5 h and increasing to 53 g/g over 72 h. The analysis of adsorption kinetics revealed that by utilizing adsorption isotherms, particularly the Langmuir isotherm, a more accurate fit to the data was achieved. This allowed for the prediction of the maximum gasoil adsorption capacity. This study aims to further develop, analyze, and utilize biocomposites made from algae-based activated carbon and polyurethane. These materials offer a sustainable and environmentally friendly approach to cleaning up oil spills.

## 1. Introduction

Oil spills are a significant concern for the environment, resulting in extensive damage to marine ecosystems and posing serious threats to wildlife and human well-being [1]. Conventional approaches to dealing with oil spills, like mechanical recovery [2], dispersants [3], and in situ burning [4], frequently prove inadequate in terms of their effectiveness, impact on the environment, and cost efficiency [5]. As a result, there is an increasing need for new materials and methods that can improve the efficiency of oil spill cleanup while reducing harm to the environment [6,7,8].

Recent advancements in the field of advanced materials, particularly in the development of nanocomposites [9,10] and polymeric foam [11,12], have demonstrated significant potential in addressing the issues related to oil spill remediation. Out of the many choices out there, hydrophobic carbon sponge nanocomposites have proven to be incredibly effective sorbents [13,14]. These materials possess impressive oil absorption capabilities, water repellent properties, and the ability to be reused, making them incredibly efficient in tackling marine oil spills [15].

Polyurethane (PU) foams have gained considerable recognition for their impressive mechanical properties, thermal and acoustic insulation, and liquid absorption capabilities [16]. When powdered, activated carbon is added to polyurethane foams; the resulting composite materials show a notable improvement in their ability to absorb oil [17]. These composites have significant potential as highly effective solutions for oil spill cleanup. They possess outstanding mechanical properties, including resistance to permanent deformation, a high Young’s modulus, and excellent resilience [15].

Water contamination by oil and chemical spills is a critical environmental issue, often occurring during the extraction, transportation, and storage of oil. Marine oil spills are particularly hazardous, as oil spreads across the water surface and can be dispersed over vast areas by wind and waves. The primary impacts include damage to wildlife and their habitats, chemical toxicity, and significant ecological alterations. The long-term environmental consequences of oil spills underscore the necessity for developing effective materials to facilitate oil removal from affected areas.

Research has focused on developing materials with substantial sorption capacities for oil spill remediation. Materials such as sugarcane bagasse, vegetable fibers, clays, and polyurethane foams have been utilized for crude oil and derivative sorption [18]. Polyurethane foams are particularly attractive for oil sorption due to their porous structure and hydrophobic polymer matrix, and they offer potential for further chemical modifications [19].

Bowen, demonstrating that adsorption occurs both on the surface and inside the foam, first studied the extraction and recovery of various inorganic and organic compounds from aqueous solutions using flexible polyurethane foams. Since then, the use of polyurethane foams in sorption processes has been extensively researched.

Recent studies have highlighted the development of polyurethane composites for environmental applications [20]. Incorporating fillers into polyurethane foams can significantly enhance properties essential for sorption, such as permeability, elasticity, chemical resistance, and hydrophobicity. These improvements can be achieved with filler concentrations typically ranging from 1% to 10% by mass.

This study aims to develop a biocomposite based on polyurethane foam and activated carbon from algae for efficient oil spill recovery. The objectives include synthesizing the biocomposite, characterizing its properties, and evaluating its performance in oil absorption tests. The research hypothesizes that the incorporation of activated carbon into the polyurethane matrix will significantly enhance the biocomposite’s oil absorption capacity, offering a sustainable solution for oil spill remediation. This study presents the incorporation of activated carbon, synthesized from readily available and low-cost algae, into the polyurethane matrix. This approach not only offers notable benefits, such as increased sorption capacity and enhanced thermal stability, but also results in a low-cost composite, making it a valuable advancement in the development of effective and economical oil spill cleaning solutions.

## 2. Materials and Methods

### 2.1. Materials

The polyurethane foams were created utilizing isocyanate (MDI, 4,4-diphenylmethane Diisocyanate: C_15_H_10_N_2_O_2_) and a polyol (Polyether Polyols: HO-(R-O)n-H). All chemicals were obtained from Pannaux Sandwich Industry in Relizane, Algeria, and were used as received.

The activated carbon derived from algae was sourced from the algae of Stadia Beach in Mostaganem, Algeria, which was treated both thermally and chemically (Table 1).

The gasoil used was provided by Arzew Refinery—Oran, Arzew, Algeria: the density at 15 °C is 0.8745, the viscosity at 20 °C is 27.441 × 10^−6^ m^2^/s, the cetane index is 49.615, and the flash point is 134 °C.

### 2.2. Biocomposite Synthesis

To synthesize the polyurethane foam (PUF) composites with algae activated carbon, we followed the following steps. Starting with the base foam, PUF-0, which was devoid of any activated carbon, the preparation utilized a one-step process. The essential aspect of the synthesis was maintaining an NCO/OH group ratio of 1.1.

Initially, the polyphenylene polyisocyanate (20 g) was thoroughly mixed with the polyol (18 g) mixture, which included water (0.3 g), silicon (0.8 g), amine (0.3 g), and pentane (1.25 g). This blending of components formed the foundational structure of PUF-0.

To create the activated foam composites, polyphenylene polyisocyanate was added to the mixture. The algae activated carbon, with a particle diameter of 200 µm (called A in this study) and 500 µm (called B in this study), was incorporated in varying amounts to produce different samples: 1.14 mass% for PUF-1, 3.34 mass% for PUF-2, and 4.41 mass% for PUF-3.

Each composite mixture was meticulously stirred to ensure a uniform distribution of the algae activated carbon particles. The mixtures were then poured into molds and allowed to cure at room temperature, resulting in the final foam composites.

### 2.3. Characterization of Samples

PUF0 and PUF3 were analyzed using various techniques to evaluate their properties and performance. SEM was used to examine the surface morphology and microstructure of the biocomposites, providing high-resolution images of the algae activated carbon particles within the polyurethane matrix [13,14,21]. The images were observed by Scanning Electron Microscopy with a Nova NanoSEM 450 (FEI, Hillsboro, OR, USA). Thermogravimetric analysis (TGA) was conducted using a TGA 2050 Thermogravimetric Analyzer (TA Instruments, New Castle, DE, USA). The thermal decomposition behavior of the samples was monitored under a nitrogen atmosphere, with the temperature increasing at a rate of 10 °C per minute from 20 °C to 600 °C. To ensure a neutral atmosphere, nitrogen was flowed for 20 min prior to heating. It was used to assess the thermal stability and composition of the biocomposites [22], determining the thermal resilience under different conditions. Density analysis (using Equations (1)–(3)) was performed to measure the bulk density of the biocomposites, relating the amount of algae activated carbon added to the composite with the resulting changes in density [16,20]. This analysis provided a better understanding of the material’s porosity and structural integrity, essential for evaluating the foam’s performance in practical applications. The density was determined by an analytical balance. Sunflower oil was used as the solvent for these experiments. The experimental device used was a SARTORIUS LA230S hydrostatic Mohr balance (Göttingen, Germany).

Apparent density was calculated using the following formula:

(1) dapparent=msampleVsample (apparent)ρwater

where

- ρ_water_: density of water;
- d_apparent_: apparent density of the sample.

Real density was calculated using the following formula:

(2) $$d_{real}=d_{air}+\frac{m_{sample\;in\;air}+d_{oil}}{A\cdot (m_{sample\;in\;air}+m_{sample\;in\;oil})}$$

where:

- d_air_: density of air;
- d_oil_: density of oil;
- d_real_: real density of the sample;
- A: corrective factor taking into account the Archimedes’ buoyancy force on the setup.

Porosity was calculated using the following formula:

(3) $$porosity=\frac{V_{pores}}{V_{total}}=1-\frac{d_{apparent}}{d_{real}}$$

### 2.4. Kinetics Study

PUF0, blank foam, and PUF–algae activated carbon composites were cut into dimensions of 10 × 10 × 10 mm and weighed to determine their oil sorption capacity in the PUF/gasoil/water systems. The samples were immersed in oil at room temperature. After a contact time, the samples were weighed again.

The oil sorption capacity was determined using Equation (1):

(4) $$q_{e}(\frac{g}{g})=\frac{m_{f}-m_{i}}{m_{i}}(\mathrm{ASTMF}726\;99)$$

where q_e_ is the oil sorption capacity (g/g), mf is the mass of the foam saturated with oil (g), and mi is the initial mass of the foam (g).

The specific testing conditions are room temperature of approximately 20 ± 3 °C and a contact time ranging from 15 to 110 min.

## 3. Results and Discussion

### 3.1. Characterization of Blank Foam and Biocomposite

The integration of activated carbon from algae into polyurethane foam significantly enhanced the oil absorption capacity of the biocomposite.

The synthesized biocomposite PUF3B displayed distinct physical and chemical properties compared to the blank foam.

#### 3.1.1. Scanning Electron Microscopy (SEM)

**Figure 1:**
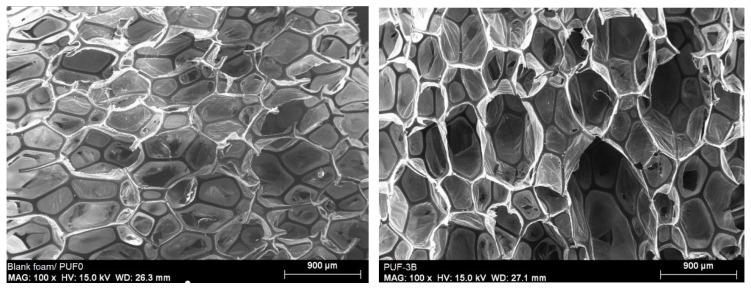
SEM images of blank foam (PUF0) and foam composite (PUF3B).

The Scanning Electron Microscopy (SEM) images in Figure 1 compare the microstructures of blank foam (PUF0) and the foam composite (PUF3B). These images provide detailed visual evidence of the differences in the cellular structure of the two materials.

The SEM images revealed a uniform distribution of activated carbon particles within the polyurethane matrix, indicating successful incorporation (Figure 1). The presence of activated carbon enhanced the surface roughness and porosity of the foam, which is beneficial for oil absorption.

The blank foam made of polyurethane (PUF0) has a more uniform and compact cellular structure, indicating potential improvements in mechanical strength and thermal insulation. This is attributed to the thicker cell walls and reduced presence of empty spaces.

In contrast, the foam composite (PUF3B) exhibits a more diverse and less compact structure. Having more voids and thinner cell walls may suggest increased flexibility or decreased heat conductivity, depending on the specific use case.

The cell size is mainly between 100 and 400 μm. Furthermore, Figure 1 shows that the addition of algae activated carbon caused some destruction of the PU cellular structure. It can be concluded that the optimal amount of algae activated carbon added to the PU structure for hydrocarbon (oil) adsorption is 4.41% by weight. Increasing the foam’s resistance enhances its ability to retain more oil within its structure.

#### 3.1.2. Thermogravimetric Analysis TGA

**Figure 2:**
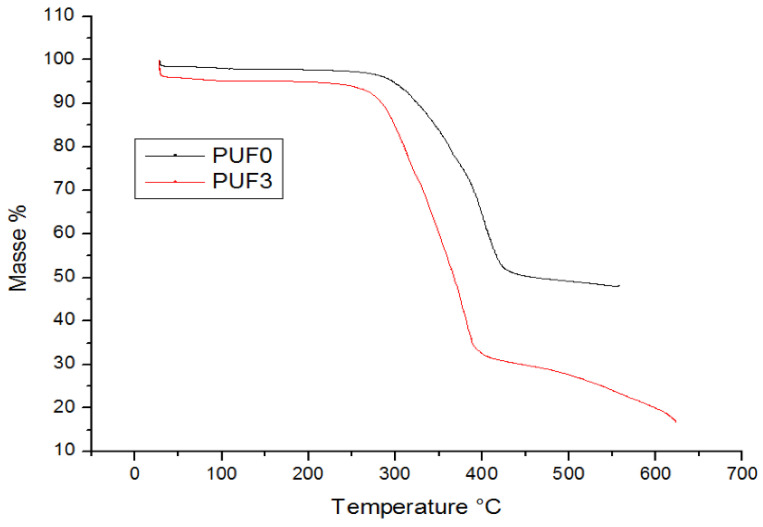
Thermogravimetric Analysis (TGA) of blank foam (PUF0) and foam composite (PUF3B).

The provided image (Figure 2) is a Thermogravimetric Analysis (TGA) graph showing the mass loss percentage of two samples, PUF0 and PUF3B, as a function of temperature (°C).

Both samples exhibit stability up to approximately 300 °C, maintaining nearly 100% of their initial mass. The onset of significant mass loss occurs at around 300 °C for both samples [1].

The TGA curves suggest that both PUF0 and PUF3B begin to decompose thermally at around 300 °C. However, PUF0 degrades more rapidly. This indicates and confirms the different compositions or the presence of more easily decomposable components in PUF0 [2].

The higher final residue in PUF0 implies that it contains more non-decomposable additives or fillers, which could affect its properties and potential applications.

The decomposition temperatures and weight loss percentages for each stage of thermal degradation are provided in Table 2 form:

#### 3.1.3. Density

**Figure 3:**
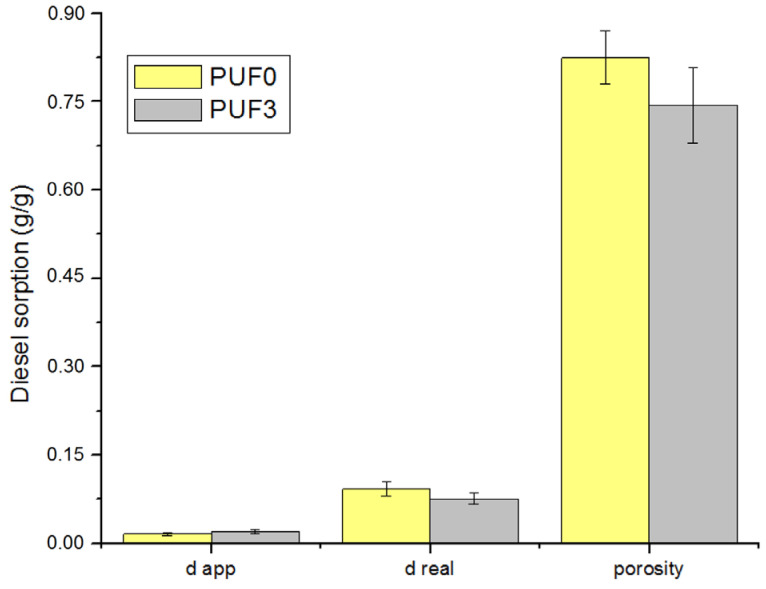
Apparent density, real density, and porosity of blank foam (PUF0) and foam composite (PUF3B).

The introduction of fillers, such as algae activated carbon, can alter the density and porosity of the foam composite; Figure 3 shows the apparent density, real density, and porosity of blank foam (PUF0) and the foam composite (PUF3B).

The study reveals that the presence of algae activated carbon in polyurethane foams significantly reduces the porosity and density. The apparent density of PUF3B (0.020 g/cm^3^) is slightly higher than that of PUF0 (0.016 g/cm^3^), indicating that the foam with algae activated carbon is denser. However, the real density of PUF0 (0.092 g/cm^3^) is slightly higher than that of PUF3B (0.076 g/cm^3^), suggesting that while the overall porosity is reduced in PUF3B, the real density is slightly lower. This difference may be due to variations in the base polymer matrix when combined with the filler [3].

Typically, polyurethane foams without fillers have porosities ranging from 70% to 90%, with PUF0′s porosity of 82.5% being within the expected range for high-porosity foams. However, with the addition of fillers like algae activated carbon, the porosity decreases by approximately 9.8%, from 82.5% (PUF0) to 74.4% (PUF3B). This decrease confirms that fillers like algae activated carbon can effectively reduce porosity by filling the foam’s voids, making the foam denser [2].

The experimental results support the typical behavior observed in filled polyurethane foams [4], validating the use of algae activated carbon as an effective filler for modifying the porosity and density of polyurethane composites.

### 3.2. Adsorption Isotherms and Kinetic Study of Gasoil

#### 3.2.1. Parameter Effects on Gasoil Adsorption Capacity

The effects of all parameters on gasoil sorption capacity were studied at different times: 1 h 30 min, 24 h, 48 h, and 72 h respectively.

- Effect of algae activated carbon content in foam on gasoil adsorption capacity

**Figure 4:**
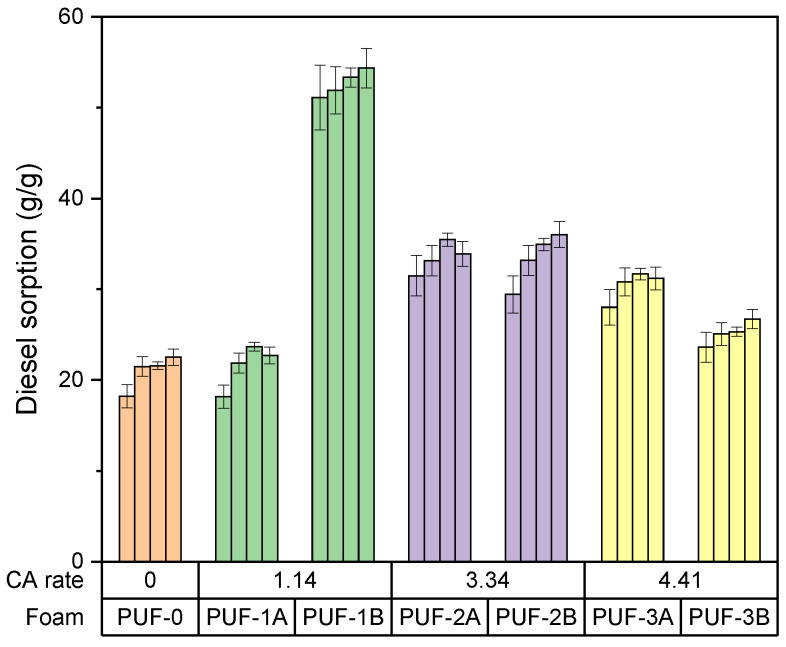
Effect of carbon content in foam on gasoil adsorption capacity.

Figure 4 depicts the effect of the carbon content in foam on the gasoil adsorption capacity.

This study reveals that the presence of algae activated carbon (CA) significantly enhances the gasoil sorption capacity of polyurethane foams.

The sorption capacity of PUF0 (blank Foam) is the lowest at approximately 20 g/g, indicating limited adsorption capability without algae activated carbon.

PUF1A and PUF1B (CA content1.14) show a sorption capacity of approximately 22 g/g, while PUF1B is significantly higher at 50 g/g.

PUF2A and PUF2B (CA content 3.34) show a sorption capacity of around 35 g/g, while PUF2B has a slightly higher capacity of approximately 45 g/g.

PUF3A and PUF3B (CA content 4.41) show a sorption capacity of around 38 g/g, and PUF3B slightly higher at around 40 g/g.

The optimal activated carbon rate for maximizing sorption capacity appears to be around 4.41, as seen in PUF3B with capacities of 53 g/g at 72 h.

In Table 3, a comparison between S (g/g) values obtained in this study and the data collected from the recent literature is reported.

- Effect of particle diameter of algae activated carbon used in foam on gasoil adsorption capacity

**Figure 5:**
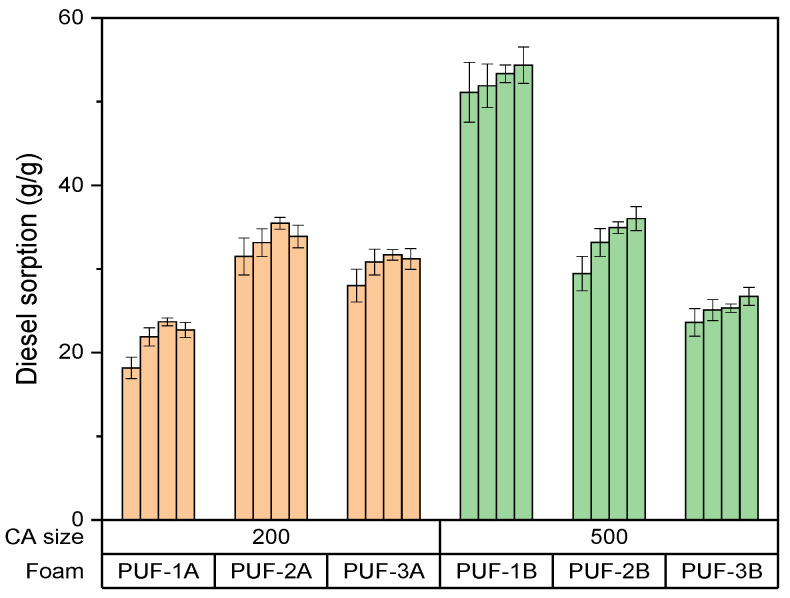
Effect of particle diameter of algae activated carbon used in foam on gasoil adsorption capacity.

Figure 5 depicts the effect of the particle diameter of algae activated carbon used in foam on gasoil adsorption capacity.

The sorption capacity of polyurethane foams is significantly enhanced by the presence and size of algae activated carbon.

The 200 µm particle size of PUF1A, PUF2A, and PUF3A exhibits a sorption capacity of approximately 22 g/g, while PUF1B, PUF2B, and PUF3B exhibit the highest sorption capacity at approximately 50 g/g. Increasing the algae carbon content initially enhances sorption capacity, but there is a slight decrease at the highest content (PUF3B). The impact of particle size on gasoil sorption capacity is significant, with larger particle sizes (500 µm) achieving approximately 50 g/g. Increasing the algae carbon content from 1.14 mass% to 3.34 mass% enhances sorption capacity, but further increasing it to 4.41 mass% shows a diminishing return in sorption capacity, especially for larger particles.

The optimal performance is achieved with a 1.14 mass% of algae carbon and a 500 µm particle size (PUF1B), achieving a high sorption capacity of approximately 50 g/g. Further optimization of the carbon content and particle size can lead to even more efficient sorption capacities for practical applications.

- Effect of time on gasoil adsorption capacity

**Figure 6:**
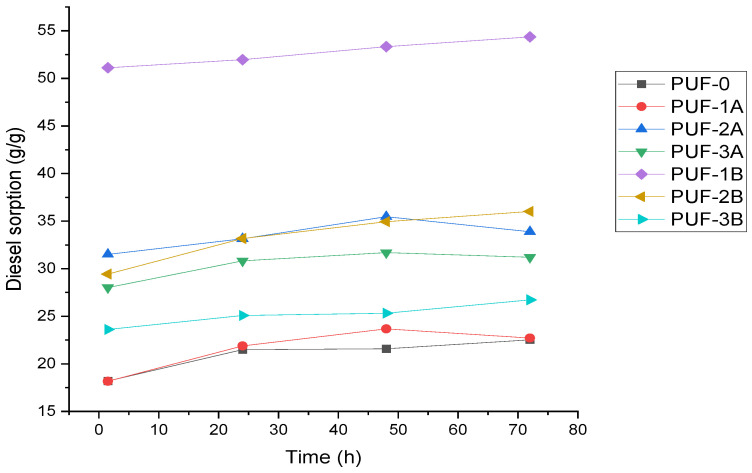
Effect of time on gasoil adsorption capacity.

Figure 6 depicts the effect of time on gasoil adsorption capacity.

This study reveals that the gasoil sorption capacity of polyurethane foams is significantly influenced by the presence and size of algae carbon particles. Samples with 500 µm size carbon particles generally exhibit higher initial gasoil sorption capacity compared to those with 200 µm size carbon particles. PUF1B, containing a 1.14 mass% of 500 µm algae carbon, starts at approximately 50 g/g at 1 h 30′ and increases slightly to 53 g/g at 72 h, demonstrating high and stable sorption capacity.

Increasing the content of algae carbon or algae activated carbon from 1.14 to 4.41 mass% enhances the gasoil sorption capacity. PUF1A, PUF2A, and PUF3A, with 200 µm particle sizes, start at 18 g/g, 28 g/g, and 32 g/g, respectively, and rise to around 25 g/g, 32 g/g, and 35 g/g at 48 h (equilibrium contact time). Similarly, PUF1B, PUF2B, and PUF3B, with 500 µm particle sizes, start at 50 g/g, 32 g/g, and 23 g/g, respectively, and increase to around 53 g/g, 35 g/g, and 25 g/g.

PUF1B (1.14 mass% of 500 µm algae carbon) exhibits the highest overall adsorption capacity of 53 g/g at 72 h, highlighting that larger particle sizes contribute to a higher initial sorption efficiency. Most samples show a gradual increase in sorption capacity over time, demonstrating good stability.

- Effect of density of algae activated carbon–foam composite on gasoil adsorption capacity

**Figure 7:**
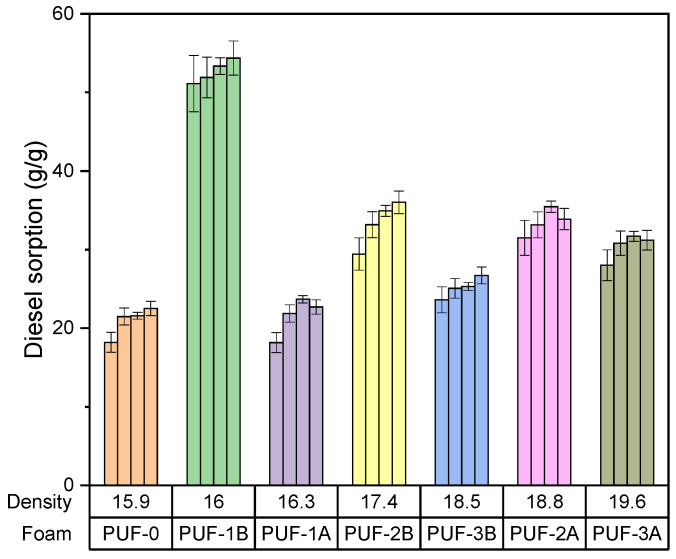
Effect of density of algae activated carbon–foam composite on gasoil adsorption capacity.

Figure 7 depicts the effect of the density of the algae activated carbon–foam composite on gasoil adsorption capacity.

The sorption capacity of polyurethane foams significantly increases with the presence of algae activated carbon. The lowest sorption capacity is observed in blank foam (PUF0), with approximately 20 g/g.

- PUF1B (1.14 mass% of 500 µm algae carbon) shows the highest initial gasoil sorption capacity at 50 g/g, indicating the impact of larger particle size on adsorption efficiency.
- PUF1A (1.14 mass% of 200 µm algae carbon) shows a sorption capacity of around 25 g/g, significantly lower than PUF1B.
- PUF2B (3.34 mass% of 500 µm algae carbon) has a sorption capacity of around 45 g/g, similar to PUF1B but slightly lower.
- PUF3B (4.41 mass% g of 500 µm algae carbon) has a gasoil sorption capacity of around 40 g/g, slightly lower than PUF2B.
- For short-term adsorption, PUF1B (1.14 mass% of 500 µm algae carbon) is most effective, with the highest initial sorption capacity of 50 g/g.

#### 3.2.2. Kinetic Study of Gasoil

- Pseudo-first-order model

**Figure 8:**
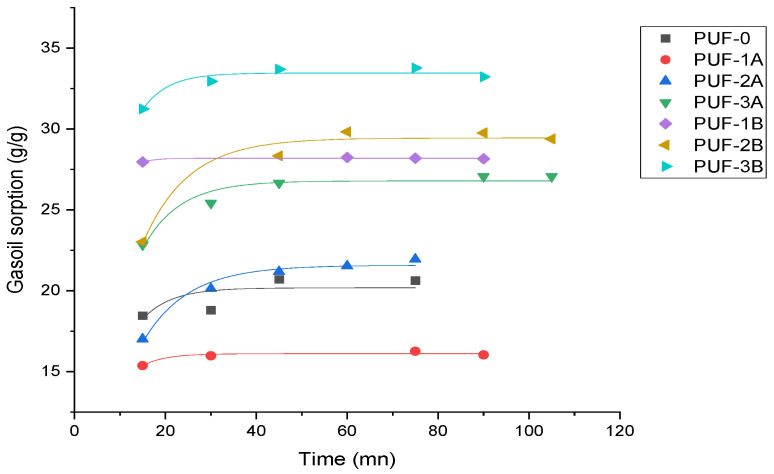
First-order model.

Figure 8 depicts the first-order-model.

With high R2 values of 0.994 and 0.976 for PUF-1B and PUF-2A, respectively, the first-order model fits most composites well. The qe results show that depending on the kind and size of algal carbon employed, there might be differences in sorption capabilities between 16.10 g/g and 33.45 g/g.

- Pseudo-second-order model

**Figure 9:**
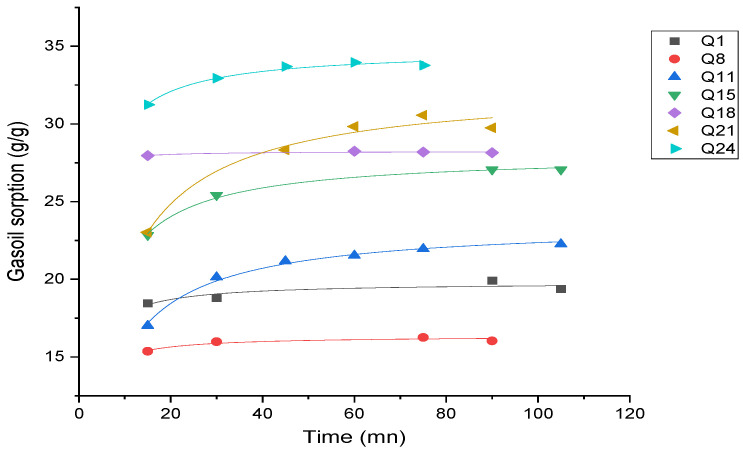
Pseudo-second-order model.

Figure 9 depicts the pseudo-second-order model.

The majority of composites also match well with the pseudo-second-order model; PUF-3A and PUF-2A have very high R2 values of 0.994 and 0.984, respectively. The qe values show how the carbon concentration of the algae affects the sorption capacity, ranging from 16.35 g/g to 34.77 g/g.

- Intraparticle diffusion model

**Figure 10:**
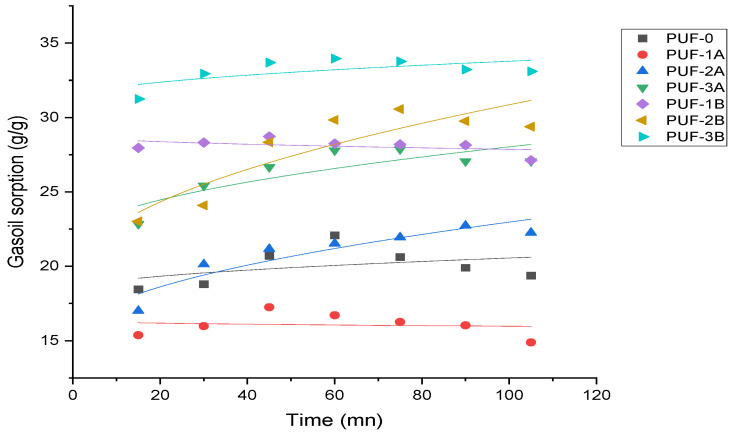
Intraparticle diffusion model.

Figure 10 depicts the intraparticle diffusion model.

The intraparticle diffusion model fits data to different degrees; for PUF-2A, the greatest R2 value is 0.848. The range of 0.037 to 1.181 is the intraparticle diffusion rate, as shown by the Kdiff values.

Table 4 provides a comprehensive comparison of the gasoil sorption capacities of various polyurethane foam (PUF) composites, analyzed using three different models: the exponential model (first-order model), the pseudo-second-order model, and the intraparticle diffusion model.

The pseudo-second-order model is the most suitable model to describe the gasoil sorption capacity of the polyurethane foam composites, according to the R2 values. Across many composites, this model continuously exhibits high R2 values, suggesting a strong match for the experimental data.

A high k value indicates that the adsorption process is driven by weaker physical forces, like van der Waals forces, leading to faster equilibrium but less strong bonds compared to chemisorption. The variation in k values among different samples reflects the speed of gasoil adsorption, with PUF1B being the most effective, influenced by the characteristics of the algal carbon used.

The most appropriate model for forecasting and examining sorption behavior in these composites is the pseudo-second-order model, which accurately depicts the kinetics of gasoil sorption in polyurethane foams with algal activated carbon to some extent.

A high k_2_ value indicates that the adsorption process is driven by strong chemical interactions, leading to the formation of stronger, more permanent bonds with the material, which enhances the overall sorption capacity and stability. The variation in k_2_ values among different composites, with PUF1B being the most effective, reflects how the composition and structure of the polyurethane foams, especially the concentration and type of algal carbon, influence the rate and strength of gasoil adsorption. The pseudo-second-order model was found to be the most suitable for describing gasoil adsorption on these composites, indicating that chemisorption, with stronger and more specific interactions, is the primary process occurring.

#### 3.2.3. Adsorption Isotherm

- Langmuir isotherm

**Figure 11:**
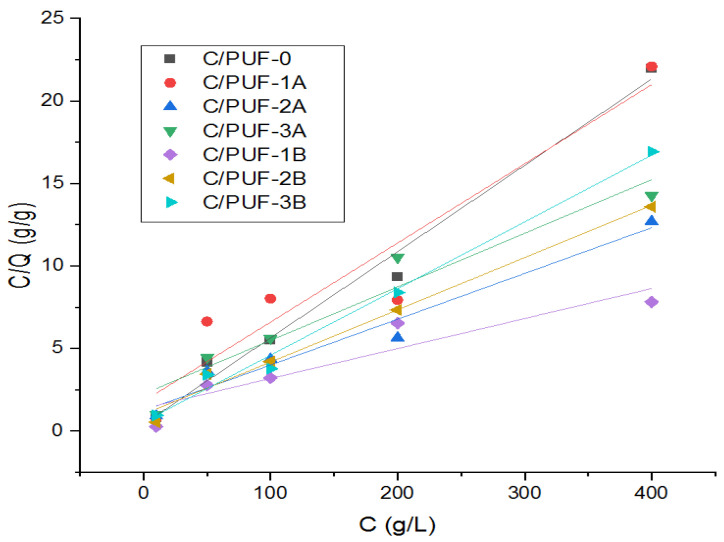
Langmuir isotherm.

Figure 11 illustrates the Langmuir isotherm correlation between several polyurethane foam (PUF) composites containing varying amounts and particle sizes of algae activated carbon. The Langmuir isotherm is often used to characterize adsorption phenomena, where the ratio C/Q (g/g) is plotted against the concentration of adsorbate in the solution C (g/L) at 1 h 30′.

- Freundlich isotherm

**Figure 12:**
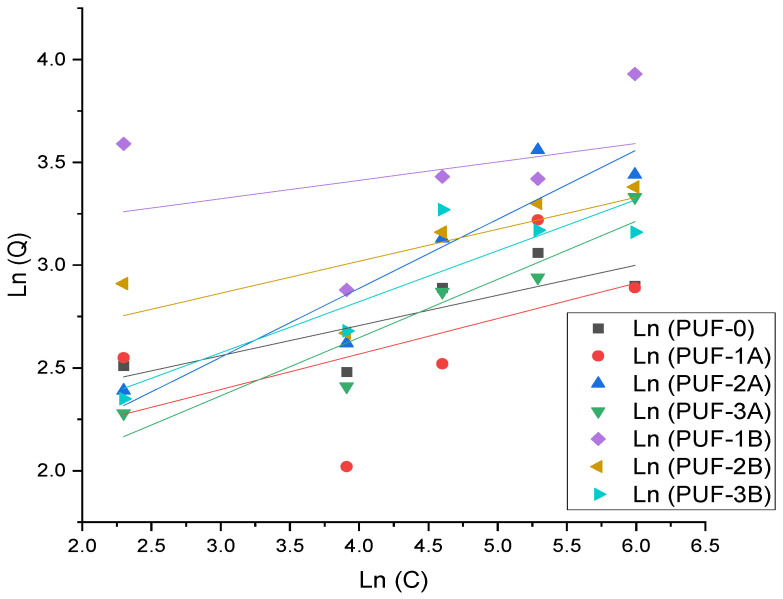
Freundlich isotherm.

The plot (Figure 12) depicts the Freundlich isotherm relationship for various polyurethane foam (PUF) composites with different amounts and sizes of algae activated carbon. The Freundlich isotherm is typically used to describe adsorption processes in heterogeneous systems, where lnQ is plotted against lnC.

Table 5 summarizes the fitting parameters for both the Langmuir and Freundlich isotherm models for the different polyurethane foam (PUF) composites. This comparison will help in understanding which model provides a better fit for each composite.

The Langmuir model is a crucial tool for understanding the adsorption behavior of polyurethane foams with algae activated carbon. It suggests that the adsorption process is better described by the Langmuir isotherm, which implies monolayer adsorption on a homogeneous surface. This model is characterized by two key parameters: Qm (maximum adsorption capacity) and Kl (Langmuir constant). The Langmuir model’s superior fit across most samples indicates that the adsorption process is dominated by monolayer coverage on a relatively homogeneous surface. This insight is crucial for designing and optimizing polyurethane foams with algae activated carbon for adsorption applications. Enhancing the uniformity of adsorption sites or optimizing the particle size of the algae carbon can lead to improved adsorption performance. The Freundlich isotherm model, which describes adsorption on heterogeneous surfaces, showed reasonable fits for some composites but was generally less effective than the Langmuir model.

## 4. Conclusions

This study developed a biocomposite based on polyurethane foam and activated carbon from algae for oil spill recovery. The biocomposite demonstrated superior oil absorption capacity, rapid uptake, and excellent reusability compared to conventional materials. The integration of renewable algal biomass into the biocomposite aligns with sustainable practices and offers an eco-friendly solution for oil spill remediation.

Structural and compositional analysis revealed a uniform distribution of activated carbon particles within the polyurethane matrix, indicating successful incorporation. The presence of activated carbon enhanced the surface roughness and porosity of the foam, which is beneficial for oil absorption with pores of 100 to 400 µm.

Density analysis showed that the apparent density of PUF3B (0.020 g/cm^3^) is slightly higher than that of PUF0 (0.016 g/cm^3^), indicating that the foam with algae activated carbon is denser. However, the real density of PUF0 (0.092 g/cm^3^) is higher than that of PUF3B (0.076 g/cm^3^), suggesting that while the overall porosity is reduced in PUF3B, the real density is slightly lower.

Thermogravimetric Analysis (TGA) was utilized to assess the thermal stability and composition of the biocomposites. Both samples exhibit stability up to approximately 300 °C, maintaining nearly 100% of their initial mass. The higher final residue in PUF0 implies the presence of more non-decomposable additives or fillers.

The presence of algae activated carbon significantly enhances the gasoil sorption capacity of polyurethane foams. Larger particle sizes (500 µm) and a higher filler content (2 g) generally result in better performance. For optimal gasoil adsorption applications, using polyurethane foams with 0.5 g of 500 µm algae carbon (PUF1B) is recommended due to its high and stable adsorption capacity. The biocomposite PUF1B exhibited the highest overall adsorption capacity, starting at approximately 50 g/g and increasing to 53 g/g over 70 h, demonstrating high and stable sorption capacity.

Isotherm models, such as the Langmuir model and Freundlich model, underscore the effectiveness of the biocomposites. The pseudo-second-order model is the most suitable for describing the gasoil sorption capacity of the polyurethane foam composites, accurately depicting the kinetics of gasoil sorption in polyurethane foams with algae activated carbon. This approach will ensure the development of optimized biocomposites that offer high performance in gasoil adsorption applications, contributing significantly to eco-friendly oil spill remediation strategies.

## Figures and Tables

**Table 1:** Properties of activated carbon derived from algae.

Iodine index	609.794 mg/g
Methylene blue index	945.26 mg/g
moisture content	1.972%

**Table 2:** Thermal degradation behavior of PUF0 and PUF3.

Decomposition Stage	First Stage		Second Stage		Final Residue	
	PUF0	PUF3	PUF0	PUF3	PUF0	PUF3
Onset Temperature (°C)	~310 °C	~300 °C	~390 °C	~370 °C	600 °C	600 °C
Endset Temperature (°C)	~390 °C	~370 °C	~450 °C	~420 °C	/	/
Weight Loss (%)	~30%	~40%	~50%	~45%	/	/
Mass Remaining at Endset (%)	~70%	~60%	~20%	~15%	~20%	~10%

This table provides a clear overview of the thermal degradation behavior of PUF0 and PUF3, highlighting their decomposition temperatures, weight loss percentages, and mass remaining after each stage.

**Table 3:** A comparison of the total diesel sorption capacity with the recent literature data.

Material	Modification Type	Sorption Capacity (g/g)	Reference
PU sponge hollow tube–graphite	Coating	20	[23]
PU–Australian palm residues	added during the polyol and isocyanate mixing	28.9	[24]
PU–textile sludge and PDM	Dip coating method	26.88	[25]
PU–Lauryl methacrylate	grafting	37.64	[16]
PU-ZnO and palmitic acid	coating	33	[26]
PU–microparticles of silica	coating	20	[27]
PU–activated carbon	coating	29.5	[27]
PU–algea activated carbon	added during the polyol and isocyanate mixing	53 after 72 h	This study

**Table 4:** Kinetic models of gasoil sorption capacities.

Equations	$Q_{t}=Q_{e}(1-exp\left(-k\cdot t\right))$			$q_{t}=\frac{q_{e}^{2}k_{2}t}{1+k_{2}q_{e}t}$			$Q_{t}=K_{diff}\sqrt{t}+C$		
PUF	q_e_ (±0.2)	k (±0.01)	R^2^	q_e_ (±0.2)	k_2_ (±0.18)	R^2^	K_diff_ (±0.03)	C (±31.23)	R^2^
0	20.17	0.16	0.532	19.80	0.042	0.798	0.221	18.341	0.160
1A	16.10	0.20	0.912	16.35	0.067	0.826	0.037	16.350	0.011
2A	21.57	0.10	0.976	23.61	0.007	0.984	0.787	21.572	0.848
3A	26.79	0.12	0.944	28.04	0.010	0.994	0.645	21.572	0.686
1B	28.19	0.31	0.994	25.25	0.223	0.987	0.093	28.792	0.185
2B	29.44	0.10	0.971	32.47	0.005	0.967	1.181	29.108	0.782
3B	33.45	0.17	0.889	34.77	0.017	0.970	0.254	31.230	0.397

**Table 5:** Adsorption isotherms.

Equations	$\frac{C}{Q}=\left(\frac{1}{Q_{m}K_{l}}\right)\cdot C+\frac{1}{Q_{m}}$			$lnQ=lnK_{f}+\frac{1}{n}lnC_{e}$		
	1/Q_m_K_l_	1/Q_m_	R^2^	lnK_f_	1/n	R^2^
PUF-0	0.420 ± 0.1	0.052 ± 0.03	0.985	2.118 ± 0.2	0.149 ± 0.01	0.647
PUF-1A	1.797 ± 11.79	0.049 ± 0.03	0.908	1.877 ± 0.1	0.172 ± 0.10	0.294
PUF-2A	1.225 ± 0.5	0.027 ± 0.04	0.905	1.544 ± 0.3	0.335 ± 0.10	0.870
PUF-3A	0.293 ± 0.1	0.093 ± 0.01	0.962	1.515 ± 0.2	0.283 ± 0.10	0.883
PUF-1B	1.362 ± 11.36	0.018 ± 0.01	0.875	2.054 ± 0.6	0.089 ± 0.10	0.109
PUF-2B	1.020 ± 11.02	0.016 ± 0.03	0.761	2.356 ± 0.3	0.15 ± 0.10	0.567
PUF-3B	0.544 ± 0.1	0.146 ± 0.02	0.852	1.829 ± 0.3	0.248 ± 0.10	0.518

## Data Availability

The original contributions presented in the study are included in the article; further inquiries can be directed to the corresponding author.
